# SKELETAL MUSCLE MITOCHONDRIAL FUNCTION AND LONGITUDINAL CHANGES IN BRAIN STRUCTURE

**DOI:** 10.1093/geroni/igae098.1059

**Published:** 2024-12-31

**Authors:** Teresa Tian, Erin Greig, Christos Davatzikos, Bennett Landman, Susan Resnick, Luigi Ferrucci

**Affiliations:** National Institute on Aging, Baltimore, Maryland, United States; National Institute on Aging, Baltimore, Maryland, United States; University of Pennsylvania, Philadelphia, Pennsylvania, United States; Vanderbilt University, Nashville, Tennessee, United States; National Institute on Aging, Baltimore, Maryland, United States; National Institute on Aging, Baltimore, Maryland, United States

## Abstract

Mitochondrial dysfunction, a hallmark of aging, has been associated with future cognitive impairment or dementia, as well as PET and blood biomarkers of Alzheimer’s disease and neurodegeneration. Whether mitochondrial dysfunction is associated with accelerated loss in brain volumes and microstructural integrity is unknown. We tested the longitudinal association between in vivo muscle mitochondrial oxidative capacity assessed via MR spectroscopy (post-exercise recovery rate of phosphocreatine, kPCr) and changes in brain MRI volumes and DTI microstructure over up to 12 years of follow-up in the Baltimore Longitudinal Study of Aging (2008-2020). In linear mixed effects models, adjusted for demographics, PCr deletion, and physical activity, higher kPCr was associated with less ventricular enlargement, less worsening in MRI-derived brain aging and Alzheimer’s disease scores, and less regional atrophy, especially the primary sensorimotor cortex, temporal white and gray matter, thalamus, occipital areas, cingulate cortex, and cerebellum (n=649). Higher kPCr was also associated with less microstructural integrity loss in white matter tracts connecting identified gray matter areas (n=598). Specific white matter tracts, including splenium of the corpus callosum and superior longitudinal fasciculus showed consistent longitudinal associations in radial diffusivity, but not axial diffusivity, which may indicate demyelination. Higher in vivo mitochondrial function is associated with less worsening in MRI-derived brain aging and Alzheimer’s disease scores, less brain atrophy in specific gray matter regions and less microstructural integrity loss in connecting white matter tracts. Future studies are warranted to investigate the role of CNS mitochondrial function in brain aging and neurodegeneration.

